# Differential microRNA expression in renal cell carcinoma

**DOI:** 10.3892/ol.2013.1460

**Published:** 2013-07-12

**Authors:** TINGTING CHENG, LINA WANG, YUYAO LI, CHEN HUANG, LINGXIA ZENG, JIN YANG

**Affiliations:** 1 Department of Medical Oncology, First Affiliated Hospital of Medical School, Xi’an Jiaotong University, Xi’an, Shaanxi 710061, P.R. China; 2 Department of Genetics and Molecular Biology, Medical School, Xi’an Jiaotong University, Xi’an, Shaanxi 710061, P.R. China; 3 Department of Statistics, Medical School, Xi’an Jiaotong University, Xi’an, Shaanxi 710061, P.R. China

**Keywords:** microRNA, renal cell carcinoma, radical nephrectomy

## Abstract

The present study aimed to detect microRNA expression levels in the tissues and sera of patients with clear cell renal cell carcinoma (ccRCC). The association of microRNA expression with ccRCC clinical pathology was analyzed, and the potential of the microRNAs as ccRCC serum markers and the significance of their expression in the clinical diagnosis, staging, prognosis and selection of new therapeutic targets for ccRCC were discussed. Specific microRNAs were selected according to the associated literature. TaqMan quantitative polymerase chain reaction (qPCR) technology was used to determine the expression levels of selected microRNAs. miR-34a, miR-224 and miR-21 were upregulated, whereas miR-141, miR-149 and miR-429 were downregulated in the ccRCC tissues (P<0.01). The expression of miR-221 and miR-211 was not significant in the ccRCC tissues (P>0.05). miR-34a, miR-21 and miR-224 were upregulated and miR-141 was downregulated in the sera of patients with ccRCC (P<0.01), while the expression of miR-149 and miR-429 was not significant (P>0.05). The serum miR-21 expression levels were significantly correlated with the clinical staging of the patients with ccRCC (P<0.05). miR-34a, miR-21 and miR-224 are upregulated in the tissues and sera of patients with ccRCC, whereas miR-141 is downregulated. miR-21 and miR-141 are associated with ccRCC and are, thus, potential ccRCC serum markers.

## Introduction

Renal cell carcinoma (RCC) accounts for 2–3% of adult malignancies and clear cell RCC (ccRCC) accounts for 80–90% of all RCCs (1). In recent years, the incidence of ccRCC has evidently increased and its mortality rate has reached 40% (2). Among ccRCC patients, ~40% are in the late stage at diagnosis due to the lack of early-stage diagnostic markers. Up to 30–40% of patients with early-stage local ccRCC suffer from relapse or metastasis, even after radical nephrectomy. ccRCC is insensitive to chemotherapy and radiotherapy; therefore, effective post-operative adjuvant therapies are lacking (3). The combined traditional immune therapy using interleukin (IL)-II and interferon does not improve the survival of patients with relapsed or advanced ccRCC (4), with five- and 10-year survival rates of <10% (5). At present, molecular-targeted therapy has been used in post-operative adjuvant therapy for patients with early-stage ccRCC or as palliative treatment for patients with advanced ccRCC (6–12). Although the objective effective rate has evidently increased, the overall survival rate among ccRCC patients is low.

The early stages of ccRCC are clinically asymptomatic. Although the advanced stages of ccRCC may have intermittent hematuria, lumbago and abdominal masses, the occurrence rate of these symptoms does not reach 15%. The patients are usually only diagnosed in the advanced stage when these symptoms are observed and the tumor has already extensively progressed and distantly metastasized. The prognosis of these patients is poor (13). Therefore, searching for tumor markers, which may be used for the early diagnosis, follow-up and clinical treatment of ccRCC patients, has become a focus of basic and clinical ccRCC research.

MicroRNAs do not encode proteins. Instead, they promote the degradation of targeted mRNA by being completely complementary to the mRNA or inhibiting the expression of the targeted mRNA by being partially complementary. MicroRNAs are involved in gene regulation at the translation level and are present in tissues, as well as in sera (14). Studies have shown that microRNAs function as oncogenes or cancer suppressor genes. They regulate the normal growth and development of organisms and are involved in tumor formation and development via the mediation of downstream target genes. MicroRNAs also function in tumor cell proliferation, apoptosis, invasion and tumor vessel formation. Studies have shown that microRNAs have specific expression profiles in the tissues and sera of patients with renal cancer (15–24). The tumor tissue may be distinguished from the adjacent normal tissue and thus patients with ccRCC may be distinguished from healthy individuals. However, no tests have confirmed the consistency of microRNA expression in the tissues and sera of patients with ccRCC. Therefore, the use of microRNA as a diagnostic marker for ccRCC is significant.

The TaqMan quantitative polymerase chain reaction (qPCR) was used in the present study to determine the microRNA expression levels in the tumor and normal tissues of patients with ccRCC and in the serum of patients with ccRCC or benign kidney lesions (BKL). The association between differential microRNA expression and gender, age, clinical stage, tumor size, invasive condition of the renal capsule and pathological classification of patients with ccRCC was also studied.

## Materials and methods

### Materials

In total, 30 pairs of ccRCC tumor tissues and normal tissue samples, 12 pre-operative serum samples from patients with ccRCC and 12 serum samples from patients with BKL were collected between April 2010 and March 2011 at the Department of Urological Surgery, First Hospital of Xi’an Jiaotong University (Xi’an, Shaanxi, China). All patients who donated tissue and ccRCC serum samples were diagnosed with ccRCC via post-operative pathology. None of these patients received pre-operative irradiation or chemotherapy.

Of the 30 tissue samples from the patients with ccRCC, 15 were from males and 15 were from females, with a mean patient age of 55 years (range, 19–74 years). Of these 30 patients, 17 were in clinical stage I and 13 were in stage II. One patient was of pathological class I, 21 were class II and eight were class III. The tumor sizes were ≤7 cm in 21 patients and >7 cm in the remaining nine patients. A total of 26 patients had invasion of the renal capsule, whereas four did not.

Of the 12 serum samples from the patients with ccRCC, eight were from males and four were from females, with a mean patient age of 54 years (range, 30–83 years). Of these 12 patients, nine were in clinical stage I, two were in stage II and one was in stage III. Three patients were of pathological class I, seven were class II, one was class III and one was class IV. The tumors were ≤7 cm in size in nine patients and >7 cm in the remaining three. Ten patients had invasion of the renal capsule, whereas two did not. The present study was conducted in accordance with the declaration of Helsinki and with approval from the Ethics Committee of Xi’an Jiaotong University. Written informed consent was obtained from all participants.

### Methods

MicroRNAs were selected for the study based on reviewed literature. To be selected, the microRNA must have been associated with ccRCC and reported in at least two studies. miR-34a, miR-21, miR-224, miR-141, miR-149, miR-429, miR-221 and miR-211 were finally selected. Total RNA was extracted from the tissue (RNAfast 200, Biotechnology Co., Ltd., Shanghai, China) and serum (mirVana™ PARIS kit, Applied Biosystems Inc., Foster City, CA, USA) samples according to the instructions of the respective extraction kits. The RNA was reverse transcribed into cDNA using the additional poly(A) tail reaction method according to the instructions of the reverse transcription kit (PrimeScript^®^ One-Step RT-PCR kit, Takara Biomedical Technology, Dalian, China). The qPCR reaction was performed according to the instructions of the qPCR kit (SYBR^®^ Premix Ex Taq™ II, Takara Biomedical Technology).

### Data analysis

Relative quantification was selected to determine the microRNA amplification. The changes in microRNA amplification were normalized with the housekeeping gene, U6. The fold changes in the microRNA of the results of the qPCR were calculated for each sample using 2^−ΔΔCt^, where ΔΔCT_1_ = (CT_miR_ − CT_U6_)_tumor_ − (CT_miR_ − CT_U6_)_adjacent_ in the tissue samples and ΔΔCT_2_= (CT_miR_ − CT_U6_)_ccRCC_ − (CT_miR_ − CT_U6_)_BKL_ in the serum samples. A change in 2^−ΔΔCt^ of >1.5 was considered an amplification or reduction of the microRNAs.

### Statistical analysis

All statistical analyses were performed using SPSS 13.0 software (SPSS Inc., Chicago, IL, USA). The differences between the tumor and adjacent tissues were tested using the paired samples t-test. The differences between the serum of patients with ccRCC and BKL were tested using an independent samples t-test or non-parametric test. The clinical correlation analysis was performed using the variance test or Kruskal-Wallis test. P<0.05 was considered to indicate a statistically significant difference.

## Results

### Tissue results

**Figure 1:**
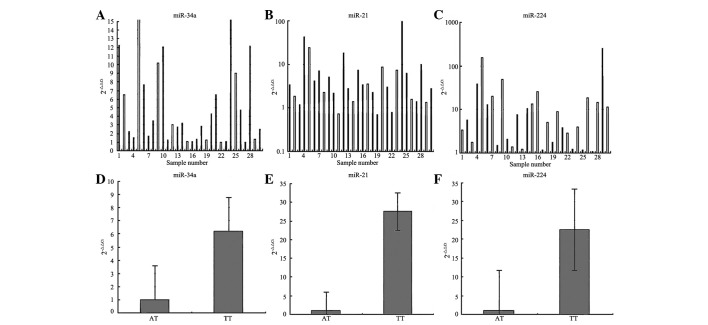
Individual (A, B, C) and general (D, E, F) relative quantification for miR-34a, miR-21 and miR-224 in tissue samples from patients with clear cell renal cell carcinoma (ccRCC). AT, adjacent tissue; TT, tumor tissue.

As shown in Fig. 1, of the 30 tissue samples, 21 expressed miR-34a, 23 expressed miR-21 and 24 expressed miR-224. The expression was upregulated in the tumor tissues compared with the normal adjacent tissues (P<0.01 for all three microRNAs). The fold changes for miR-34a, miR-21 and miR-224 were 6.2, 22.5 and 27.5, respectively.

**Figure 2:**
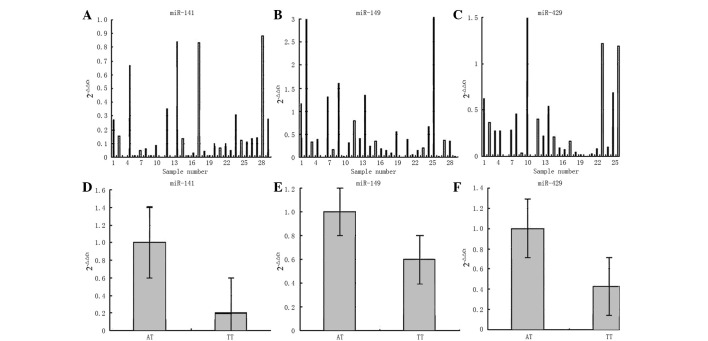
Individual (A, B, C) and general (D, E, F) relative quantification for miR-141, miR-149 and miR-429 in tissue samples from patients with clear cell renal cell carcinoma (ccRCC). AT, adjacent tissue; TT, tumor tissue.

As shown in Fig. 2, of the 30 tissue samples, 26 expressed miR-141, 21 expressed miR-149 and 22 expressed miR-429. The expression was downregulated in the tumor tissues compared with the normal adjacent tissues (P<0.01 for all three microRNAs). The fold changes for miR-141, miR-149 and miR-429 were 6.3, 1.7 and 2.4, respectively.

**Figure 3:**
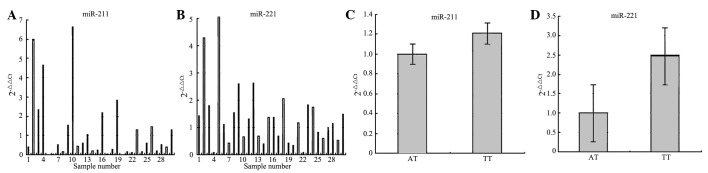
Individual (A, B) and general (C, D) relative quantification for miR-211 and miR-221 in tissue samples from patients with clear cell renal cell carcinoma (ccRCC). AT, adjacent tissue; TT, tumor tissue.

As shown in Fig. 3, no significant differences were observed in the expression of miR-211 and miR-221 in the tumor and normal adjacent tissues (P=0.339 and P=0.271, respectively). Of the 30 tissue samples, miR-211 expression was upregulated in seven and downregulated in 18, whereas miR-221 was upregulated in eight and downregulated in seven.

### Serum results

**Figure 4:**
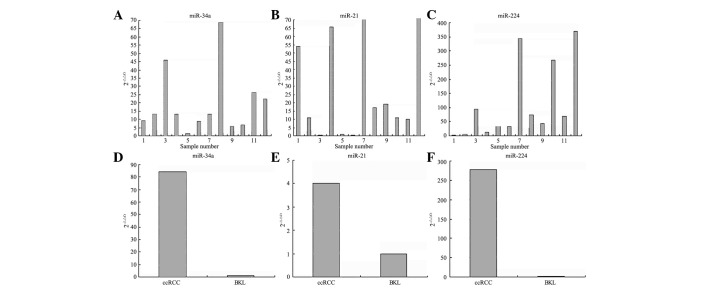
Individual (A, B, C) and general (D, E, F) relative quantification for miR-34a, miR-21 and miR-224 in serum samples from patients with ccRCC compared with those with BKL. ccRCC, clear cell renal cell carcinoma; BKL, benign kidney lesion.

As shown in Fig. 4, of the 12 serum samples, 11 expressed miR-34a, 12 expressed miR-21 and nine expressed miR-224. The microRNA expression was upregulated in the serum of patients with ccRCC compared with those with BKL (P<0.01 for all three microRNAs). The fold changes for miR-34a, miR-21 and miR-224 were 84, 277 and 4, respectively.

**Figure 5:**
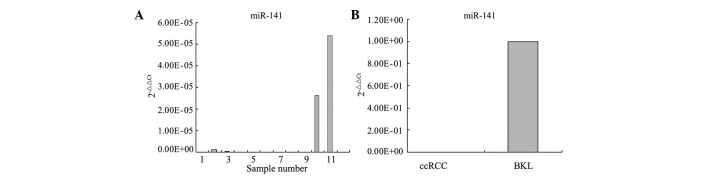
Individual (A) and general (B) relative quantification for miR-141 in serum samples from patients with ccRCC compared with those with BKL. ccRCC, clear cell renal cell carcinoma; BKL, benign kidney lesion.

As shown in Fig. 5, all 12 serum samples expressed miR-141. The expression was downregulated in the serum of patients with ccRCC compared with those with BKL (P<0.01). The fold change for miR-141 was 5.8×10^7^.

**Figure 6:**
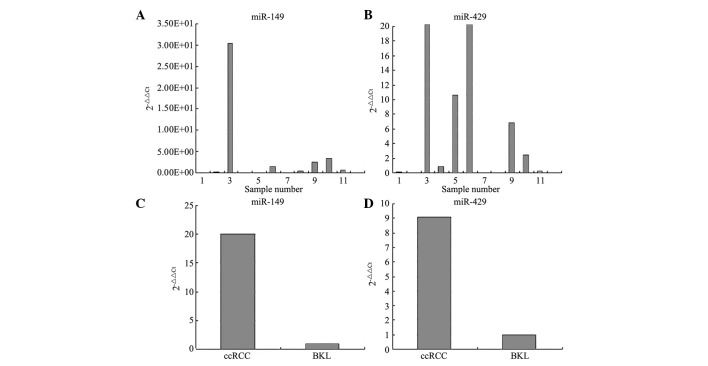
Individual (A, B) and general (C, D) relative quantification for miR-149 and miR-429 in serum samples from patients with ccRCC compared with those with BKL. ccRCC, clear cell renal cell carcinoma; BKL, benign kidney lesion.

As shown in Fig. 6, no significant differences were observed in the expression of miR-149 and miR-429 in the serum of patients with ccRCC and BKL (P=0.754 and P=0.657, respectively).

### Correlation between microRNA expression and clinicopathological characteristics in ccRCC patients

The correlations between microRNA expression in the 30 tissue samples from the ccRCC patients and the clinicopathological features are presented in Table I. No significant correlations were observed between microRNA expression and gender, age, clinical stage, pathological grade, tumor size or invasive condition of the renal capsule of tissue samples from patients with ccRCC.

**Figure 7:**
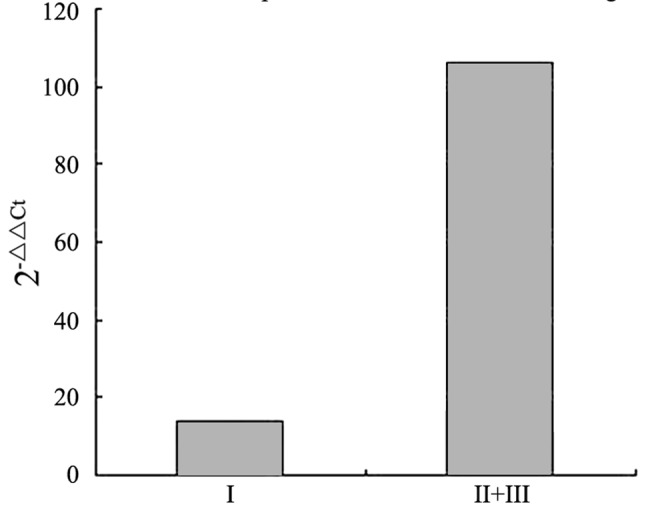
Correlation between miR-21 and clinical stage in serum samples of clear cell renal cell carcinoma patients (P<0.05).

The correlation between microRNA expression in the 12 serum samples from patients with ccRCC and the clinicopathological features is shown in Table II. The correlation between miR-21 expression and the clinical stage was significant (P<0.05; Fig. 7). The correlation between miR-224 expression and gender was significant (P<0.05; Fig. 7) No significant correlation was observed between the expression of the other microRNAs and gender, age, clinical stage, pathological grade, tumor size and invasive condition of renal capsule of the serum samples from the patients with ccRCC.

## Discussion

As a solid tumor, ccRCC is resistant to chemotherapy and radiotherapy. The main therapeutic method for local ccRCC is radical nephrectomy, which has a five-year survival rate of 60–80%. However, the five-year survival rate of patients with metastatic ccRCC is <10% due to the lack of effective post-operative adjuvant therapy. The early stage of ccRCC is clinically asymptomatic; thus, the majority of patients with ccRCC already have distant metastasis with clinical symptoms upon diagnosis. At present, no tumor marker for early ccRCC diagnosis has been identified. Therefore, the present study attempted to identify a tumor marker that may be used to diagnose the early stage of ccRCC based on the quantity of microRNA expression in the tumors and sera of ccRCC patients.

The study showed that the expression levels of miR-34a, miR-224 and miR-21 were significantly higher in ccRCC tumor tissues compared with the adjacent tissues (P<0.01). The expression levels of miR-141, miR-149 and miR-429 were significantly lower in the tumor tissues compared with the adjacent tissues (P<0.01). The results were consistent with those of previous studies (15–24). However, the microRNA expression levels had no correlation with the clinical data of the patients with ccRCC in the present study. Moreover, the expression levels of miR-34a, miR-224 and miR-21 were significantly higher in the sera of the patients with ccRCC compared with those with BKL (P<0.01), while the expression levels of miR-141 were significantly lower in the sera of the ccRCC patients compared with those with BKL (P<0.01). The expression profiles of miR-34a, miR-224, miR-21 and miR-141 in the sera of patients with ccRCC were consistent with those of the corresponding tumor tissue samples. The miR-21 expression levels in the serum of the patients with ccRCC were correlated with the patients’ clinical stage (P<0.05) and miR-224 expression levels were correlated with gender The present study showed that miR-34a, miR-224, miR-21 and miR-141 are potential ccRCC tumor markers. The study demonstrated that the expression of miR-224 and miR-141 in the sera of patients with ccRCC was significantly different. However, no significant correlation was observed between microRNA expression and the clinicopathological characteristics of the patients, with the exception of the significant correlation between serum miR-21 expression and ccRCC clinical stage due to the smaller sample size. Further studies with greater sample sizes are required to confirm microRNA as a ccRCC serum marker.

Studies on ccRCC have demonstrated that microRNA expression differs significantly between ccRCC tumors and the adjacent normal tissues (15–24). In 2010, Chen (15) demonstrated that microRNA expression was significantly different in the ccRCC serum. However, the consistency of microRNA expression in the tumor tissues and sera of ccRCC patients has not yet been demonstrated. The present study is the first to demonstrate the significance of the expression of miR-34a, miR-224, miR-21 and miR-141 in the tissues and sera of patients with ccRCC, with coinciding expression trends.

In 2008, Chen *et al*(14) demonstrated that microRNAs are resistant to RNase, indicating that they are maintained in the serum and plasma of humans and other animals. Serum microRNAs are normally derived from circulatory hemocytes, whereas tumor-related microRNAs are derived from circulatory hemocytes, as well as tumor tissues and tumor-affected tissues. Moreover, Mitchell *et al*(25) further demonstrated that microRNAs from tumor cells enter the blood circulation and that microRNA expression levels are correlated with tumor size. The microRNAs result from the initiating tumor cell secretions and are potential tumor serum markers. Wei *et al*(26) and Tomimaru *et al*(27) considered that miR-21 may be used as a tumor serum marker to diagnose hepatocellular carcinoma and non-small cell lung cancer. Certain studies have shown that miR-141 has the potential as a tumor serum marker for prostatic and colorectal cancer (28–31).

Inhibiting miR-21 expression arrests the cell cycle of tumor cells and induces apoptosis in the ccRCC cell line. Consequently, the tumor cell invasiveness and metastatic capacity would also decline. The results of a study by Zaman *et al* also showed that the average survival time of ccRCC patients with low miR-21 expression was more than five years and that the five-year survival rate of ccRCC patients with high miR-21 expression was only 50%. In addition, the miR-21 expression levels increased with the increasing ccRCC pathological grade (32).

Another study showed that miR-224 expression in ccRCC tumor tissues was four-fold higher compared with a control group (P<0.01) (33). The study suggested that miR-224 acts directly on the DIO1 UTR in ccRCC. The specific change in the tumors is negatively correlated with the concentration of intracellular DIO1 and T3. Induced miR-224 expression in Caki-2 cells causes a significant decline in DIO1 mRNA levels (P<0.01) (33).

miR-141 expression is downregulated significantly in ccRCC, which is associated with the epithelial-mesenchymal transition (EMT) (34). EMT causes the loss of cell adhesion and promotes cancer cell metastasis, including ccRCC metastasis. Liu *et al*(21) suggested that the loss of expression of miR-149, miR-200c and miR-141 causes the activation of oncogenes [KCNMA1, LOX, vascular endothelial growth factor A (VEGFA) and SEMA6A]. The expression of the miR-200 family (miR-200a^*^, miR-200b, miR-200c and miR-141) was negatively correlated with VEGFA, and SEMA6A was the direct target gene of miR-141.

miR-141 and miR-21 are involved in the progression of ccRCC. In the present study, no significant correlation was observed between the other microRNAs and the ccRCC clinicopathological data in the tissue and serum from patients with ccRCC. However, serum miR-21 expression was significantly correlated with the clinical staging of ccRCC and serum miR-224 expression was significantly correlated with gender due to the small sample size. Therefore, miR-141, miR-224 and miR-21 are potential ccRCC serum markers.

## Figures and Tables

**Table I:** Correlation between the expression of microRNAs and clinicapathological characteristics in tissue samples from patients with ccRCC.

		miR-34a		miR-224		miR-141		miR-149		miR-429		miR-21	
							
Characteristics	n	2^−ΔΔCt^ ± SD	P-value	2^−ΔΔCt^ ± SD	P-value	2^−ΔΔCt^ ± SD	P-value	2^−ΔΔCt^ ± SD	P-value	2^−ΔΔCt^ ± SD	P-value	2^−ΔΔCt^ ± SD	P-value
Gender													
Male	15	5.59±7.79	0.702^a^	9.07±10.71	0.534^b^	0.19±0.25	0.941^a^	0.91±1.05	0.051^b^	0.31±0.31	0.234^a^	12.82±25.33	0.885^b^
Female	15	6.76±8.75		35.96±70.81		0.20±0.28		0.28±0.29		0.55±0.70	5.38±5.82		
Age (years)													
≤50	9	7.87±9.82	0.467^a^	31.81±81.52	0.528^a^	0.35±0.39	0.331^b^	0.65±0.58	0.809^a^	0.22±0.22	0.182^a^	12.94±31.00	0.603^b^
>50	21	5.45±7.49		18.53±33.86		0.13±0.16		0.57±0.92		0.51±0.62		7.46±9.99	
Clinical stage													
I	16	5.17±8.22	0.479^a^	32.82±68.97	1.000^b^	0.19±0.31	0.889^a^	0.44±0.50	0.237^a^	0.28±0.33	0.115^a^	6.74±11.10	0.339^b^
II	14	7.33±8.24		10.75±13.09		0.20±0.21		0.78±1.07		0.60±0.70		11.80±24.55	
Pathological grade													
I+II	22	5.68±7.01	0.590^a^	20.16±52.34	0.685^a^	0.20±0.28	0.909^a^	0.51±0.66	0.334^a^	0.37±0.55	0.357^a^	8.55±19.85	0.792^a^
III	8	7.54±11.21		29.01±52.39		0.19±0.22		0.84±1.18		0.58±0.56		10.62±15.02	
Tumor size (cm)													
≤7	13	5.70±9.10	0.785^a^	21.00±41.72	0.891^a^	0.13±0.19	0.214^a^	0.32±0.36	0.180^b^	0.26±0.35	0.153^a^	7.77±12.17	0.735^a^
>7	17	6.54±7.63		23.67±59.31		0.25±0.30		0.81±1.01		0.55±0.65		10.13±22.44	
Renal capsule invasion													
No	4	12.26±14.21	0.111^a^	5.40±6.45	0.486^a^	0.13±0.13	0.595^a^	0.58±0.68	0.972^a^	0.40±0.55	0.934^a^	28.75±44.67	0.088^a^
Yes	26	5.24±6.80		25.15±55.04		0.21±0.28		0.60±0.85		0.43±0.56	6.08±9.18		

a ANOVA;

b Kruskal-Wallis test.

ccRCC, clear cell renal cell carcinoma.

**Table II:** Correlation between the expression of microRNAs and clinicapathological characteristics in serum samples from patients with ccRCC.

		miR-34a		miR-224		miR-141		miR-21	
					
Characteristic	n	2^−ΔΔCt^ ± SD	P-value	2^−ΔΔCt^ ± SD	P-value	2^−ΔΔCt^ ± SD	P-value	2^−ΔΔCt^ ± SD	P-value
Gender									
Male	8	24.7±22.3	0.212^a^	161.4±104.9	0.011^b^	0.1E-5±0.2E-5	0.606^b^	38.8±60.0	0.860^a^
Female	4	9.2±5.7		15.4±16.3		0.1E-6±0.7E-7		32.8±31.8	
Age (years)									
≤50	5	21.1±27.0	0.827^a^	27.2±29.9	0.056^a^	0.1E-6±0.6E-7	0.533^b^	29.7±28.4	0.703^a^
>50	7	18.4±14.6		173.8±147.7		0.1E-5±0.2E-5		41.9±64.2	
Clinical stage									
I	9	20.6±22.7	0.758^a^	69.6±79.9	0.229^a^	0.1E-5±0.2E-5	0.480^a^	13.7±16.7	0.013^b^
II+III	3	16.3±5.3		242.2±198.1		0.1E-6±0.1E-7		106.1±58.8	
Pathological grade									
I	3	41.4±29.9	0.116^b^	57.1±48.1	0.782^b^	0.1E-6±0.1E-7	1.000^b^	23.7±27.6	0.629^a^
II+III+IV	9	12.2±8.1		131.3±149.8		0.1E-5±0.2E-5		41.2±57.2	
Tumor size (cm)									
≤7	9	21.5±21.7	0.566^a^	100.1±129.5	0.596^a^	0.4E-6±0.9E-6	0.770^b^	28.5±29.7	0.926^b^
>7	3	13.5±12.6		150.5±168.5		0.2E-5±0.4E-5		61.6±97.1	
Renal capsule invasion									
No	2	3.3±3.2	0.216^a^	40.6±2.6	0.667^b^	0.1E-6±0.0	0.644^a^	10.1±12.8	0.441^a^
Yes	10	22.7±19.9		127.2±143.0		0.1E-5±0.2E-5		42.1±54.2	

a ANOVA;

b Kruskal-Wallis test.

ccRCC, clear cell renal cell carcinoma.
